# The making of a pest: Insights from the evolution of chemosensory receptor families in a pestiferous and invasive fly, *Drosophila suzukii*

**DOI:** 10.1186/s12864-016-2983-9

**Published:** 2016-08-17

**Authors:** Paul V. Hickner, Chissa L. Rivaldi, Cole M. Johnson, Madhura Siddappaji, Gregory J. Raster, Zainulabeuddin Syed

**Affiliations:** Department of Biological Sciences & Eck Institute for Global Health, University of Notre Dame, Notre Dame, IN 46556 USA

**Keywords:** Olfaction, Gustation, Odorant receptors, Gustatory receptors, Niche utilization, Adaption, Molecular evolution, Episodic selection

## Abstract

**Background:**

*Drosophila suzukii* differs from other *melanogaster* group members in their proclivity for laying eggs in fresh fruit rather than in fermenting fruits. Olfaction and gustation play a critical role during insect niche formation, and these senses are largely mediated by two important receptor families: olfactory and gustatory receptors (*Ors* and *Grs*). Earlier work from our laboratory has revealed how the olfactory landscape of *D. suzukii* is dominated by volatiles derived from its unique niche. Signaling and reception evolve in synchrony, since the interaction of ligands and receptors together mediate the chemosensory behavior. Here, we manually annotated the *Ors* and *Grs* in *D. suzukii* and two close relatives, *D. biarmipes* and *D. takahashii*, and compared these repertoires to those in other *melanogaster* group drosophilids to identify candidate chemoreceptors associated with *D. suzukii’*s unusual niche utilization.

**Results:**

Our comprehensive annotations of the chemosensory genomes in three species, and comparative analysis with other *melanogaster* group members provide insights into the evolution of chemosensation in the pestiferous *D. suzukii*. We annotated a total of 71 *Or* genes in *D. suzukii*, with nine of those being pseudogenes (12.7 %). Alternative splicing of two genes brings the total to 62 genes encoding 66 *Ors*. Duplications of *Or23a* and *Or67a* expanded *D. suzukii’*s *Or* repertoire, while pseudogenization of *Or74a*, *Or85a,* and *Or98b* reduced the number of functional *Ors* to roughly the same as other annotated species in the *melanogaster* group. Seventy-one intact *Gr* genes and three pseudogenes were annotated in *D. suzukii*. Alternative splicing in three genes brings the total number of *Grs* to 81. We identified signatures of positive selection in two *Ors* and three *Grs* at nodes leading to *D. suzukii*, while three copies in the largest expanded *Or* lineage, *Or67a*, also showed signs of positive selection at the external nodes.

**Conclusion:**

Our analysis of *D. suzukii*’s chemoreceptor repertoires in the context of nine *melanogaster* group drosophilids, including two of its closest relatives (*D. biarmipes* and *D. takahashii*), revealed several candidate receptors associated with the adaptation of *D. suzukii* to its unique ecological niche.

**Electronic supplementary material:**

The online version of this article (doi:10.1186/s12864-016-2983-9) contains supplementary material, which is available to authorized users.

## Background

Chemoreception, broadly encompassing olfaction and gustation, is essential to a number of insect life history traits such as host detection and discrimination, mate location, and predator avoidance. Chemoreception in insects is largely mediated by two divergent protein families, olfactory receptors (Ors) and gustatory receptors (Grs). A third family described in 2009 by Benton et al. [1] as the ionotropic receptors (Irs) has been implicated in multiple sensory modalities, including chemosensation [2]. Insect chemoreceptors (Ors and Grs) are seven transmembrane proteins expressed on the surface of chemosensory neurons housed in hair-like structures called sensilla [3, 4]. The genome of *Drosophila melanogaster* contains 60 *Ors* encoding 62 proteins through alternative splicing [4, 5], and each *Or* is expressed in a specific sub-set of olfactory receptor neurons (ORNs), with very few exceptions. All ORNs expressing the same *Or* merge into a single glomerulus [6]. While the basic principles and mechanisms of olfaction remain conserved across phyla [3, 7, 8], insect *Ors* have little homology to *Caenorhabditis elegans* or vertebrates, and the membrane topology is quite distinct [9]. Moreover, all canonical *Ors* are co-expressed with a single noncanonical olfactory receptor co-receptor (*Orco*), and together appear to define the response characteristic of an ORN [10]. The sense of taste in *D. melanogaster* is defined by 60 *Grs* encoding 68 proteins through alternative splicing [5]. In contrast to Ors, there is no clear evidence for a non-canonical co-receptor, and the membrane topology remains poorly defined [11].

The number of chemoreceptors often varies widely among insects, broadly reflecting their environment and function [3]. For example, the tsetse fly, *Glossina morsitans*, is estimated to have 40–46 *Ors* and 11–14 *Grs* [12, 13], while the red flour beetle, *Tribolium castaneum*, has 259 *Ors* and 220 *Grs* [14] (*Tribolium* Genome Sequencing Consortium 2008), and the honey bee, *Apis mellifera*, has 163 *Ors* and only 10 functional *Grs* [15]. The largest chemoreceptor repertoires (over 350 Ors) are reported in eusocial insects, such as ants [16]. In *Drosophila, Or* repertoires reflect the niche specialization patterns, such that a restricted spectrum of host/diet choice can be correlated with changes in chemoreceptor repertoire, such as specific losses and/or duplications in a set of receptors [17–21]. These changes can be further correlated with structural changes to the peripheral olfactory apparatus such as an altered number of specialized sensilla/ORNs [22–24]. Since, signaling and reception evolve in synchrony and in parsimony [25], an overall understanding of both these aspects will provide insights into the chemosensory basis of host utilization.

The recent (*Drosophila* 12 Genomes Consortium, Drosophila modENCODE Project) sequencing and subsequent annotation of multiple *Drosophila* spp. provides us with an excellent opportunity to connect the natural history of drosophilids [26] with the evolutionary history of chemosensation [27]. Recently, a member of the *melanogaster* group, *Drosophila suzukii* (Matsumura), has gained immense attention due to its invasion of the western hemisphere from its original endemic zone of South East Asia and emergence as a serious economic pest. A reduction in the yield of berry and soft fruit crops in newly invaded areas of North America and Europe are reported to reach as high as 80 % in the absence of any management practices, although a current and comprehensive economic assessment is lacking [28, 29].

Among the Drosophilidae, comprising over 1,500 known species [30], *D. suzukii* is one of only a few *Drosophila* with a highly evolved serrated ovipositor [31] that enables gravid females to pierce the skin of fresh fruits and lay their eggs inside the flesh. Though *D. suzukii* has been recognized as a pest of cherries in Japan since 1931, they were found infesting strawberries and cranberries in California, USA in 2008 [29]. They have since been discovered in at least a dozen states in the USA, as well as areas of Canada, Mexico, Italy, Spain and France [28, 29, 32]. We recently conducted a comprehensive analysis of the suite of volatile organic chemicals (VOCs) that define the unique olfactory landscape of *D. suzukii*, and compared it with that of *D. melanogaster* [33]. We demonstrated that *D. suzukii’s* unique attraction to fresh fruits may be associated with the distinctive volatile repertoire originating from the host fruit-fly associated yeast complex. Recent studies are providing exciting insights into the complex interactions of *D. Suzukii* with yeast and fruits [34].

Here, we explored the role of olfaction and gustation in *D. suzukii’s* unique ecological niche. We first manually annotated the Ors and Grs in the recently sequenced *D. suzukii* genomes [35, 36], and two closely related species, *D. biarmipes* and *D. takahashii* (Drosophila modENCODE Project), herein collectively referred to as the *suzukii-takahashii* clade. The latter two species occur in geographically overlapping regions with *D. suzukii* [30] but are mostly saprophytic and do not have the pointed ovipositor that enables them to lay eggs in fresh fruits [31]. We then compared these repertoires to those in six other previously annotated *melanogaster* group *Drosophila* [5, 19, 37]. Following our earlier comprehensive analysis of ligand repertoires for *D. suzukii* [33], we present the associated chemoreceptor repertoire that together defines *D. suzukii’*s unique ability to exploit diverse niches, and in turn pose a serious threat to fruit crops. This study further adds to ongoing efforts in understanding the chemosensory basis of host and mate finding in *D. suzukii* [33, 34, 38–40].

## Methods

### Manual curation of *Or* and *Gr* repertoires

*D. suzukii* gene models were manually curated based on the *D. melanogaster Or* and *Gr* annotations in FlyBase version FB2015_03 [41]. In short, *D. melanogaster* peptide sequences were used to screen the *D. suzukii* genome scaffolds using tBLASTn analysis in SpottedWingFlyBase v1.0 (last accessed on 4 September, 2015) [35]. To help predict start and stop codons, and exon-intron boundaries, scaffold regions containing putative chemoreceptors were aligned with their homologous *D. melanogaster* coding sequences (CDS) in MultAlin [42]. Where exon-intron boundaries were ambiguous, intron donor and accepter sites were evaluated using the splice site prediction tool [43] on the Berkeley Drosophila Genome Project web site (http://www.fruitfly.org/seq_tools/splice.html). Complementary strands were generated using the Reverse Compliment tool in the Sequence Manipulation Suite [44] (http://www.bioinformatics.org/sms/rev_comp.html), and coding sequences were translated using the ExPASy translate tool [45]. The *D. suzukii Or* and *Gr* annotations were then used to screen the *D. biarmipes* (Dbia_2.0, GCA_000233415.2) and *D. takahashii* (Dtak_2.0, GCA_000224235.2) genome assemblies with the methods described for *D. suzukii* using the BLAST tools on the National Center for Biotechnology Information (NCBI) web server. The *D. biarmipes* and *D. takahashii* genome assemblies were generated and made publicly available by the Drosophila ModENCODE project and the Baylor College of Medicine-Human Genome Sequencing Center (BCM-HGSC).

### Gap filling and sequence validation

#### Gap filling

We filled gaps in the genome scaffolds that prevented the building of complete gene models using the sequence read archive (SRA) databases in NCBI. In those gene models where this method failed, PCR and capillary sequencing were used to fill the gaps.

#### Validation of duplications

Two approaches were used to evaluate duplications. When possible, tandem repeats were confirmed by amplifying and sequencing a region spanning the proximal ends of the duplicates. However, when the copies were greater than ~4,000 nucleotides apart or on a different scaffold we sequenced the individual genes.

#### Validation of pseudogenes

Predicted pseudogenes were resequenced to confirm the predictions from the initial tBLASTn analysis for *D. suzukii*, *D. takahashii* and *D. biarmipes.*

Genomic DNA (gDNA) for resequencing was extracted from the strains used for genome sequencing that are presently available at the UC San Diego Drosophila Stock Center: *D. suzukii* (stock # 14023–0311.03), *D. biarmipes* (stock # 14023–0361.10) and *D. takahashii* (stock # 14022–0311.13). A cetyltrimethylammonium bromide (CTAB) protocol [46] was modified for the extraction of genomic DNA from insects. Ten adult flies (5 males and 5 females) were ground with a pestle in 1.5 ml microcentrifuge tubes containing 200 μl 2 % CTAB solution (100 mM Tris HCl pH 8.0, 10 mM EDTA, 1.4 M NaCl, and 2 % CTAB). Samples were incubated for 5 min at 65 °C, followed by the addition of 200 μl chloroform and mixing by inverting 10 times. Samples were then centrifuged for 5 min at 13,000 x g. The aqueous phase was removed and placed in a new tube containing 200 μl isopropanol, mixed by inverting 10 times, and centrifuged for 5 min at 13,000 x g. The supernatant was poured off, 500 μl of 70 % ethanol was added, and the sample was centrifuged for 5 min at 13,000 x g. The supernatant was removed and the pellet was allowed to dry at room temperature for 15 min. DNA was resuspended in 50 μl deionized water, and all samples were normalized to 50 ng/μl using a Nanodrop ND-2000 (ThermoScientific, USA).

Primers flanking the gaps were designed using the Primer3plus program [47]. PCR was carried out in 50 μl reaction volumes using GoTaq® reagents (Promega). Each reaction contained a final concentration of 0.2 μM of each primer, 1.0 units of *Taq* polymerase and 2 ng/μl of genomic DNA. The thermal cycle included an initial denaturation of 94 °C for 2 min, 35 cycles of 94 °C for 1 min, 58 °C for 1 min, and 72 °C for 2 min, with a final extension time of 5 min at 72 °C. PCR products were visualized with agarose gel electrophoresis using SYBR® Safe gel stain (ThermoScientific). PCR products were cleaned-up using the Wizard® SV Gel and PCR Clean-Up System (Promega). Sequencing was performed using the ABI 3730xl (Life Technologies) and BigDye® chemistry (Life Technologies) at the University of Notre Dame Genomics Core Facility. Genes with sequence gaps filled using the SRA databases or by PCR and sequencing were suffixed with “fixSRA” or “fixPCR”, respectively. Nucleotides that were fixed based on SRA or PCR are in bold or underlined, respectively (Additional file 1: Table S1-S6).

### Gene nomenclature

*Ors* and *Grs* were named based on homology to *D. melanogaster* using standard *Drosophila* community gene nomenclature [48]. Each gene was prefixed with ‘D’ and the first three letters of the specific epithet (Dsuz, Dbia or Dtak), and named based on a combination of phylogenetic and reciprocal BLASTp analyses with the *D. melanogaster* annotated protein database in FlyBase version FB2015_03 [41]. Duplications were suffixed with a unique numeral (e.g. *DsuzOr23a-1* and *DsuzOr23a-2*). Splice variants were predicted solely on genomic sequence (no transcript evidence) and suffixed using the capital letter designation in accordance with the homologous splice variant in FlyBase for *D. melanogaster* (e.g. *DmelOr69aA* and *DsuzOr69aA*). However, where novel splice variants were predicted, splice variants were designated based on their order on the scaffold rather than homology to *D. melanogaster*.

Pseudogenes were suffixed with ‘P’, and are defined here as genes with a mutated start codon, premature stop codon, or frameshift mutation leading to loss of ≥20 % of the original protein and ≥1 transmembrane domain [19] compared to the *D. melanogaster* homolog. The number of transmembrane domains was predicted using the topology prediction program, OCTOPUS [49]. Pseudogenes that were not excessively degraded were reconstructed for phylogenetic analysis by repairing mutated start codons, exon-intron boundaries or frameshift mutations to a functional state based on an intact homolog in the *suzukii* or *takahashii* subgroup. Repaired nucleotides are in lowercase in Table S1. All genes other than pseudogenes and partial gene models are assumed to be functional and are referred to here as intact. We refer to a lineage as lost when pseudogenizations or deletions (no apparent vestiges) resulted in the absence of at least one intact gene in one of 47 *Gr* or 54 *Or* orthologous groups (OGs) present in the *melanogaster* group as defined by Almeida et al. 2014 [50] (see Additional file 2: Table S3 and S4).

Comparisons were made to the previously annotated chemoreceptor repertoires of *D. melanogaster* [5], *D. ananassae*, *D. erecta*, *D. sechellia*, *D. simulans*, and *D. yakuba* [19, 37]. To better characterize lineages that were lost in the *suzukii*-*takahashii* clade, we screened the genomes of six additional *Drosophila* genome assemblies (*D. bipectinata*, *D. elegans*, *D. eugracilis*, *D. ficusphila*, *D. kikkawai*, and *D. rhopaloa*), generated and made publicly available by the BCM-HGSC, using the methods described above. Evolutionary inferences were based on phylogeny reconstruction by Chiu et al. [35], while divergence times were based on earlier estimates [36]. Reconciliation of gene trees with the species tree for the expanded lineages was performed using the parsimony-based method in NOTUNG v2.8.1.6 [51]. Gene trees were estimated using Mega version 6 [52] where the maximum likelihood approach with the Jones, Taylor, Thornton (JTT) substitution model [53], a Gamma distribution (+G) with five discrete categories, and complete deletion of gaps was implemented. The edge weight thresholds were 0.9 and based on bootstrap support following 500 iterations, while the loss and duplication costs were 1.0 and 1.5, respectively. No branches were collapsed for NOTUNG analysis.

### Measures of divergence

Two proxies were used to describe divergence, the percent of identical amino acids in a peptide sequence alignment to *D. melanogaster* (%ID) and the ratio of nonsynonymous (*dN*) to synonymous (*dS*) substitution rates (*dN*/*dS*). %ID was calculated using Clustal Omega [54] on the European Bioinformatics Institute (EMBL-EBI) web server [55]. Nonsynonymous and synonymous substitution rates were calculated using the Nei and Gojobori method [56] implemented in SNAP v2.1.1 [57] (http://www.hiv.lanl.gov/content/sequence/SNAP/SNAP.html). *D. melanogaster* was used as an outgroup for *dN* and *dS* calculations for all three species (i.e. *suzukii*-*melanogaster, biarmipes*-*melanogaster,* and *takahashii-melanogaster*). Differences were determined using paired (between species) and unpaired (*Ors* vs *Grs*) Wilcoxon Signed-Rank tests with the MASS package [53] in the R statistical environment.

### Tests for positive selection

Positive selection acting on a small proportion of sites is often hard to detect using the ratio of nonsynonymous to synonymous substitution rates across the entire length of a gene (*dN*/*dS*). Therefore, we used the adaptive branch-site random effects likelihood (aBSREL) approach [52] to identify signatures of diversifying selection at the codon level within a phylogenetic framework comprised of 9 species in the *melanogaster* group: *D. ananassae*, *D. biarmipes*, *D. erecta*, *D. melanogaster*, *D. sechellia*, *D. simulans*, *D. suzukii*, *D. takahashii* and *D. yakuba. Or* sequences for *D. ananassae*, *D. erecta*, *D. sechellia*, *D. simulans* and *D. yakuba* were from Guo and Kim [37] while *Gr* sequences were kindly provided by Michael Ritchie (University of St Andrews, UK). Only functional genes were used in the positive selection analysis.

Peptide sequences of homologous chemoreceptors (gene sets) were aligned in MAFFT v7 using the Blosum62 scoring matrix, a gap penalty of 1.53, and the G-INS-1 refinement method [58]. Each alignment was visually inspected and manually edited, when necessary, and used to estimate a phylogeny for each homologous gene set. The maximum likelihood approach with the Jones, Taylor, Thornton (JTT) substitution model [59] and a Gamma distribution (+G) with five discrete categories, and complete deletion of gaps was implemented in Mega version 6 [60]. Codon alignments were generated using PAL2NAL [61]. The aBSREL method [52] was implemented in HyPhy [62], where all internal and external nodes were tested for signatures of diversifying selection using likelihood ratio tests (LRTs). The Holm-Bonferroni method was used to control the familywise error rate for multiple tests within a gene set [63], whereas the Benjamini-Hochberg false discovery rate method was used for corrections across all gene sets [64]. Chemoreceptors showing positive selection based on the aBSREL method were further tested by using the stringent M1–M2 models of the codeml program in PAML [65]. Values >0.95 from Bayes empirical Bayes (BEB) method were considered sites under diversifying selection [66].

### Phylogenetic analysis

Phylogenies were estimated for Ors and Grs to help reconstruct evolutionary events and to assist in the naming of the genes. Peptide sequences of *D. suzukii*, *D. biarmipes*, *D. takahashii* and *D. melanogaster* ≥ 360 aa (Ors) or ≥ 340 (Grs) in length were multiply aligned using MUSCLE v3.8.31 [67]. Maximum likelihood trees were inferred using the PROTGAMMA model of protein substitution, JTT matrix, and 500 bootstrap replications in RAxML v.8 [68]. RAxML analysis was conducted on the CIPRES Science Gateway and XSEDE [69]. Figures were prepared using the FigTree program for visualization and annotation of phylogenetic trees [70]. The *Or* and *Gr* trees were rooted with *Orco* and *Gr21a*, respectively. The aligned peptide sequences files (Phylip) and phylogenetic tree files (Nexus) for both the OR and Gr families are in the additional files (Additional files 3, 4, 5 and 6)

### Scanning electron microscopy (SEM)

Freshly emerged *D. suzukii* were placed in acetone for at least 24 h until they could be processed by scanning electron microscopy (SEM). After undergoing critical point drying, flies were mounted both dorsally and ventrally on carbon tape attached to an aluminum stub mount, and coated with 4 μM of iridium using a Cressington 208 HR sputter coater (Cressington Scientific Instruments, Watford, UK) in conjunction with the Cressington MTM 20 thickness monitor. Images were taken with a FEI-Magellan 400 FESEM (FEI, Hillsboro, OR, USA).

## Results

### Chemosensory organs and receptor repertoires

**Fig. 1:**
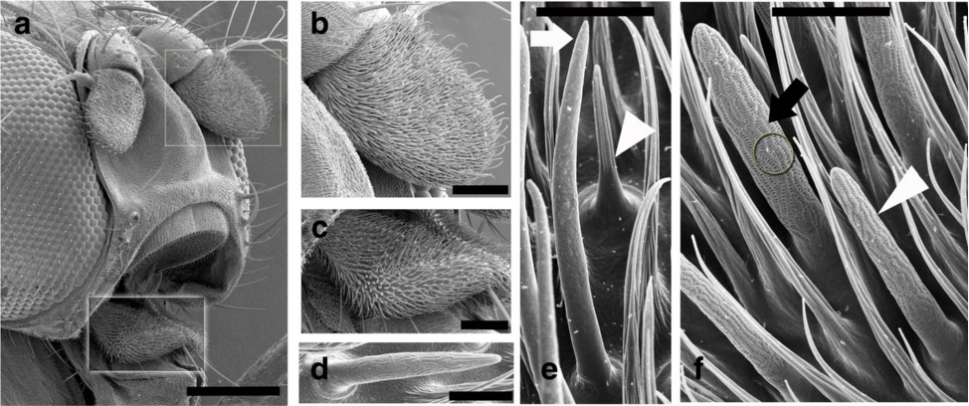
Olfactory structures in *D. suzukii* viewed under SEM. An adult head with antenna and maxillary palp, highlighted in the insets. **a** Magnified antenna (**b**) and a maxillary palp (**c**). Palps have only one type of multiporous basiconic sensillum type (**d**). Antennal surface shows: long pointed trichoid sensillum (white arrow) and distinct coeloconic (arrow head) (**e**); multiporous basiconic sensilla (black arrow points to the large basiconic and short basiconic are indicated by white arrow head) (**f**). Scale bar is 200 μM in (**a**), 50 μM in (**b**) and (**c**); 5 μM for (**d**), (**e**) and (**f**)

Scanning Electron Microscopy (SEM) of the olfactory organs in *D. suzukii* revealed striking morphological similarity to the well-defined *D. melanogaster* structures (Fig. 1) [71, 72]. Maxillary palps were adorned with a single class of olfactory sensilla, basiconic (Fig. 1c, d), whereas an additional two types, trichodea and coeloconic, are seen on the antenna (Fig. 1e). One unusual feature we noted in the large basiconic class was the presence of two distinct pore patterns. The single pattern reported earlier in *D. melanogaster* (Fig. 1f; circle) [71, 72] was observed *in D. suzukii*, but we also noted an additional unique pore pattern (Fig. 1f; circle).

**Fig. 2:**
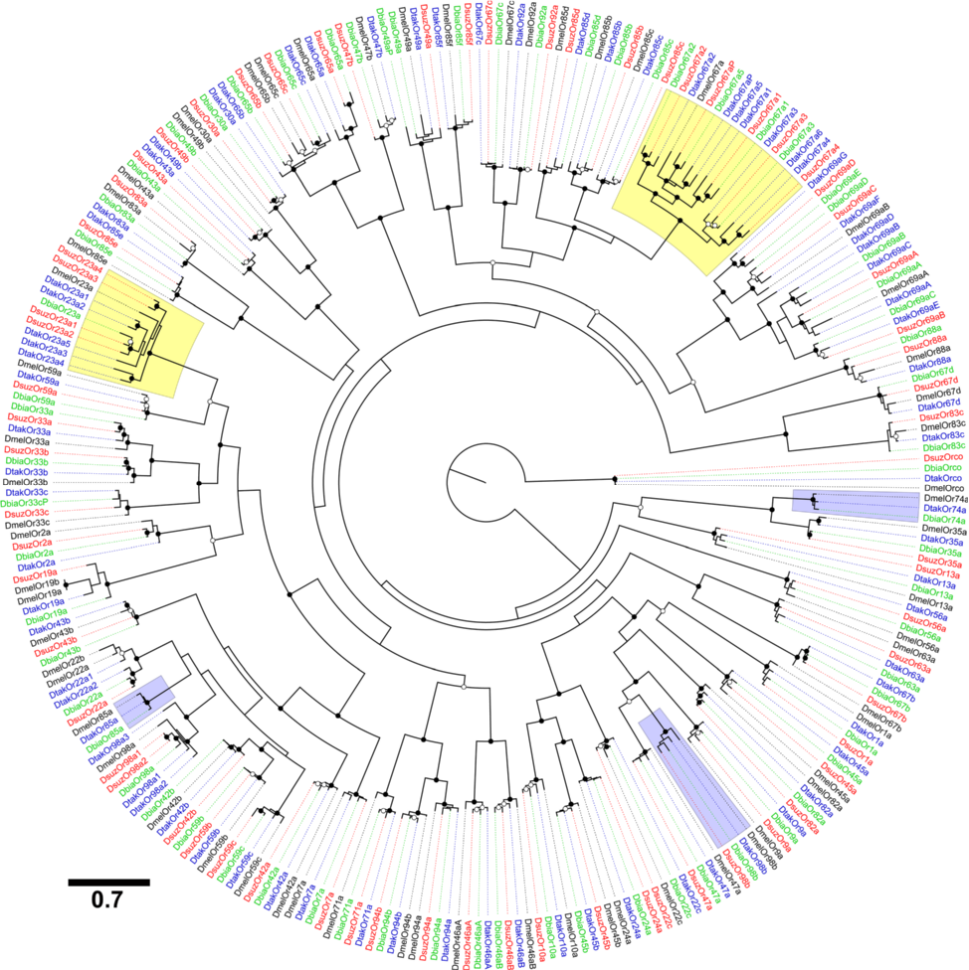
Phylogenetic analysis of *Ors* in four *Drosophila* using a Maximum Likelihood method. The evolutionary history was inferred by using the Maximum Likelihood method based on the JTT matrix-based model [59]. The tree was constructed using RAxML under the JTT model of substitution with NNI topology search [68], based on an amino acid alignment by MUSCLE [67]. Branch support was estimated using 500 bootstrap replications. Expanded and lost lineages in D. *suzukii* are highlighted in yellow and blue, respectively

Next, we annotated the *Ors* and *Grs* from the genome assemblies of *D. suzukii* and two closely related members, *D. biarmipes* and *D. takahashii*. A summary of the *Or* and *Gr* repertoires, along with those previously annotated in *D. melanogaster*, are reported in Table 1. Phylogenetic relationships among the *Ors* in these four species are represented in Fig. 2, illustrating several clade specific and species specific expansions. The total number of *Or* loci ranged from 64 in *D. biarmipes* to 71 in *D. suzukii and D. takahashii*. However, pseudogenizations reduced the number of functional *Or* gene*s* to 62, 60, and 70 in *D. suzukii*, *D. biarmipes* and *D. takahashii*, respectively. We predicted alternative splicing in two *Or* genes (*Or46a* and *Or69a*) in all three species, the same genes with splice variants in *D. melanogaster* [5]. *Or46a* encodes two splice variants that are moderately conserved, with percent identity of *D. suzukii* to *D. melanogaster* ranging from 80.7 to 83.4 % (Additional file 1: Table S1). Conversely, *Or69a* is predicted to encode four to seven splice variants in the *suzukii-takahashii* clade, compared to only two isoforms in *D. melanogaster*. The number of functional genes in *D. suzukii* and *D. biarmipes* is roughly the same as *D. melanogaster*, whereas *D. takahashii*, with 70 genes, is more than the 66 predicted in *D. ananassae,* the largest *Or* repertoire among the *melanogaster* group *Drosophila* annotated prior to this study.

**Table 1:** Summary of the chemoreceptor repertoires in *D. suzukii* (Dsuz), *D. biarmipes* (Dbia)*,* and *D. takahashii* (Dtak), along with those previously annotated in *D. melanogaster (*Dmel)

	ORs				GRs			
	Dsuz	Dbia	Dtak	Dmel*	Dsuz	Dbia	Dtak	Dmel*
Loci	71	64	71	62	74	74	88	62
Functional genes	62	60	70	60	71	74	82	60
Pseudogenes	9	4	1	2	3	0	6	2
Genes w/splice variants	2	2	2	2	3	3	3	3
Splice variants	6	7	9	4	13	12	12	11
Total functional proteins	66	65	77	62	81	83	91	68

Total functional proteins include predicted splice variants

*Data from [5, 77, 97–99]

**Fig. 3:**
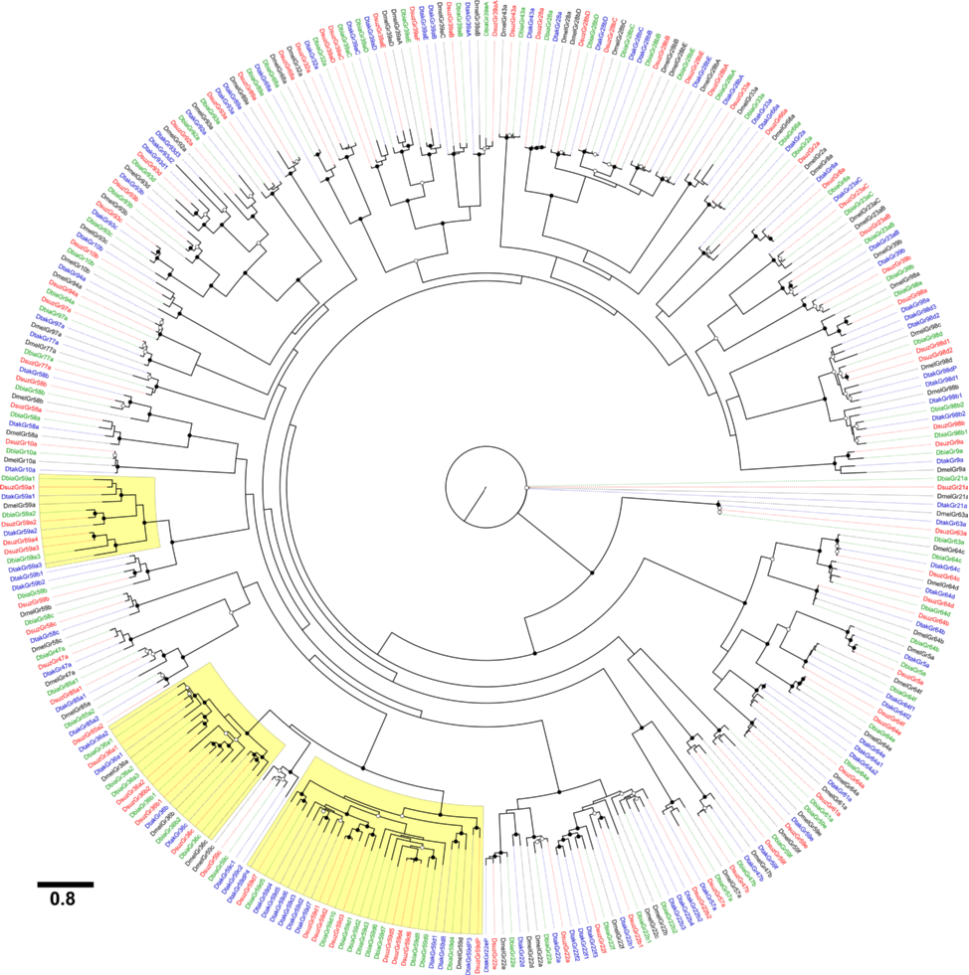
Phylogenetic analysis of *Grs* in four *Drosophila* using a Maximum Likelihood method. The evolutionary history was inferred by using the Maximum Likelihood method based on the JTT matrix-based model [59]. The tree was constructed using RAxML under the JTT model of substitution with NNI topology search [68], based on an amino acid alignment by MUSCLE [67]. Branch support was estimated using 500 bootstrap replications. Expanded lost lineages in D. *suzukii* are highlighted in yellow

We predicted a total of 74 *Gr* genes in *D. suzukii,* of which 71 are functional and three are pseudogenes, while 74 intact genes and no pseudogenes were predicted in *D. biarmipes*, and 88 genes were predicted in *D. takahashii* of which six are pseudogenes (Table 1). Phylogenetic relationships among the *Grs* in four species showed several unique expansions (Fig. 3). In *D. suzukii*, three genes encode 13 splice variants, bringing the total to 81 functional *Grs* (Table 1). *D. suzukii’s* repertoire of *Grs* is nearly identical to *D. biarmipes*, which has 74 genes encoding 83 Grs. While these two *Gr* repertoires are larger than any other *Drosophila* annotated thus far, *D. takahashii’s* repertoire is even larger with 82 intact genes encoding 91 Grs (Table 1).

The number of introns in *Ors* and *Grs* was consistent with those in *D. melanogaster*, with the exception of *Gr85a. D. suzukii*, *D. biarmipes* and *D. takahashii* each have two copies of *Gr85a*, and *Gr85a-1* has one intron while *Gr85a-2* has two introns. Furthermore, the peptide sequences are notably shorter (374–381 aa) than *Gr85a* in *D. melanogaster* (397 aa). The functional state of *Or42a* in both *D. suzukii* and *D. biarmipes* was initially unclear due to an unusually long first intron. *Or42a* resides on two different scaffolds in both species where it is fragmented in the 1^st^ intron. Attempts to amplify and sequence the gene region were unsuccessful. *Or42a* in *D. takahashii* has a large first intron (2511 nucleotides) compared to *D. ananassae* (66 nucleotides) and *D. melanogaster* (185 nucleotides), so next we examined *Or42a* in other *melanogaster* group genomes and found that the first intron is also large in *D. kikkawai* (4,475), and on two different scaffolds in the *D. eugracilis* assembly. Consequently, failure to amplify the gene could have been due to the size of the amplicon. Screening of the SRA from transcriptome sequencing by Chiu et al. [35], however, shows that *Or42a* is being transcribed in *D. suzukii*; therefore, we considered *Or42a* intact in *D. suzukii* and *D. biarmipes*.

### Evolutionary events

#### Expansions and losses

**Fig. 4:**
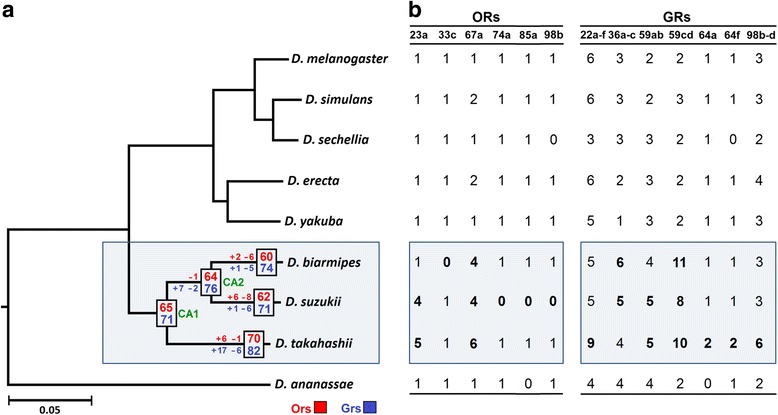
Evolutionary events in the *suzukii-takahashii* clade chemoreceptor families. **a** Evolutionary changes in the number of functional *Or* and *Gr* genes in the clade derived from common ancestors, CA1 and CA2. **b** The number of intact genes in the expanded and lost lineages in the clade (shaded) is shown in bold, and is compared with the number in six other *melanogaster* group *Drosophila*. Phylogeny adapted from Chiu et al. [35]

Gene tree reconciliation revealed complex birth-and-death evolutionary patterns, wherein the *suzukii* and *takahashii* subfamilies (Fig. 4a and b; shaded box) underwent changes in copy numbers in a subset of *Ors* and *Grs* as they diverged from their common ancestor (CA1 in Fig. 4a). The later split of *D. suzukii* and *D. biarmipes* from CA2 was accompanied by similar changes. Three *Or* lineages, *Or74a*, *Or85a* and *Or98b* were lost in *D. suzukii* but were functional in *D. biarmipes* and *D. takahashii,* while *Or33c* was lost in *D. biarmipes*, and none were lost in *D. takahashii* (Fig. 4). Based on previous annotations, and the screening of five additional *melanogaster* group genomes, the loss of *Or74a* is unique to *D. suzukii,* while *Or85a* was lost independently in *D. ananassae* and *D. suzukii.*

The two largest expansions in the *D. suzukii* and *D. takahashii Or* lineages were *Or23a* and *Or67a* (Fig. 4b; Additional file 7: Figure S1). *D. suzukii* and *D. takahashii* have four and five copies of *Or23a*, respectively, while *D. biarmipes* has only one (Fig. 4b). Four intact and one *Or67a* pseudogene were found in *D. suzukii*, while four intact copies were found in *D. biarmipes*, and six copies plus a pseudogene were found in *D. takahashii* (Fig. 4b). The *Gr* lineages showed by far the largest expansions in the *suzukii-takahashii* clade compared to all of the annotated *melanogaster* group *Drosophila.* Four lineages were expanded in *D. suzukii*, two in *D. biarmipes*, and six in *D. takahashii* (Fig. 4a and b; Additional file 7: Figure S1). One lineage, *Gr59cd*, was expanded in all three members of the *suzukii-takahashii* clade, whereas *Gr36a-c* was uniquely expanded in the *D. suzukii* and *D. biarmipes.* The only other shared expansion was between *D. suzukii* and *D. takahashii* for *Gr59ab*. The largest number of unique expansions in the *suzukii-takahashii* clade was in *D. takahashii* and includes *Gr22a-f*, *Gr64a*, *Gr64f* and *Gr98b-d*. Interestingly, no *Gr* lineages were lost in any of the three species annotated in the present study.

Next, we used the parsimony-based gene tree reconciliation method in NOTUNG v2.8.1.6 [51] to analyze the two largest expanded lineages in both *Ors* and *Grs*. Among the *Ors*, *Or23a* duplicated several times, and the common ancestor to the *suzukii-takahashii* clade probably had three copies, indicating that *D. biarmipes* lost two copies while *D. suzukii* and *D. takahashii* gained one and two copies, respectively (Additional file 7: Figure S1). The expansion of the *Or67a* lineage was already present prior to the *suzukii-takahashii* split, except for one later duplication in *D. takahashii* (Fig. S1). The two largest expanded *Gr* lineages were *Gr59a* and *Gr59d* in all three species. The *Gr59a* duplication pattern was comparable to *Or67a*, whereas *Gr59d* showed by far the most complex pattern of evolution resulting in 27 total copies in the three species (Fig. S1).

#### Divergence

Having annotated the genomes of three species that include the pest, *D. suzukii*, we estimated divergence and selection in the chemosensory receptor families using the percent of identical amino acids to homologous *D. melanogaster* peptide sequences (%ID) and the ratio of nonsynonymous (*dN*) to synonymous (*dS*) substitution rates (*dN*/*dS)* (Table 2)*.* The *dN/dS* ratios in *Ors* ranged from 0.0125 in *DsuzOrco* to 0.3670 in *DbiaOr19a* (mean of three species = 0.111), while the %IDs ranged from 44.72 % in *DbiaOr67a*-2 to 98.77 % in *DsuzOrco* (mean = 81.9 %). The %ID of Grs ranged from 33.06 % in *DbiaGr59a*-3 to 99.55 % in *Gr21a* (mean = 74.8 %). The *Gr* dN/dS ratios ranged from 0.002 in *Gr21a* to 0.370 in *Gr10b* (mean = 0.138). These low *dN*/*dS* values imply that both chemoreceptor families have evolved under strong purifying selection. These values are larger than the reported genome wide estimates of 0.095 for X chromosome genes and 0.090 for autosomal genes [35],. Differences in the means between species, based on paired Wilcoxon Signed-Rank tests, are shown in Table 2. Comparisons between *dN*, *dS*, and *dN/dS* of *Ors* and *Grs* using unpaired tests showed that *Grs* are more divergent than *Ors* in all three species (Table 3).

**Table 2:** Substitution rate analysis of *Ors* and *Grs*

		species			*p*-values		
Genes	Parameter	Dsuz	Dbia	Dtak	suz-bia	suz-tak	bia-tak
*Ors*	*dN*	0.094	0.103	0.089	0.023	0.001	<0.001
	*dS*	0.936	1.009	0.945	0.007	0.790	0.048
	*dN/dS*	0.102	0.104	0.093	0.679	0.006	0.009
*Grs*	*dN*	0.146	0.152	0.141	0.000	0.004	<0.001
	*dS*	1.036	1.039	1.018	0.373	0.489	0.707
	*dN/dS*	0.141	0.142	0.131	0.003	0.008	0.014

Differences in mean nonsynonymous (*dN*) and synonymous (*dS*) substitution rates, and *dN*/*dS* are indicated by asterisks

**Table 3:** Differences in substitution rates between *Ors* and *Grs*. Mean synonymous (*dS*) and nonsynonymous (*dN*) substitution rates and ratios (*dN/dS*) for *Ors* and *Grs* using *D. melanogaster* as an outgroup

	Dsuz			Dbia			Dtak		
Parameter	*Ors*	*Grs*	*p*-value	*Ors*	*Grs*	*p*-value	*Ors*	*Grs*	*p*-value
*dN*	0.094	0.146	0.004	0.103	0.152	0.005	0.089	0.141	0.001
*dS*	0.936	1.036	0.924	1.009	1.039	0.681	0.945	1.018	0.623
*dN/dS*	0.102	0.141	0.005	0.104	0.142	0.002	0.093	0.131	0.001

Mean *dN* and *dN/dS* was greater for *Grs* suggesting that, overall, *Grs* were more divergent than *Ors* in all three species

Genes with the highest and lowest *dN/dS* values in *D. suzukii* provide insights into highly divergent or conserved functions. Among the most conserved *Ors*, *Orco* tops the list, followed by *Or47a*, *Or92a*, *Or42b* and *Or24a*, and whereas *Gr21a*, *Gr28a*, *Gr28bB*, *Gr63a* and *Gr64c* were the most conserved *Grs*. Most divergent *Ors* were *Or19a*, *Or23a*, *Or69aA*, *Or65a* and *Or33a* and the *Grs* included *Gr10b*, *Gr93d*, *Gr92a*, *Gr85a* and *Gr22c* (Additional file 2: Tables S1 and S2). This trend was comparable in *D. takahashii* and *D. biarmipes*.

#### Selection

**Fig. 5:**
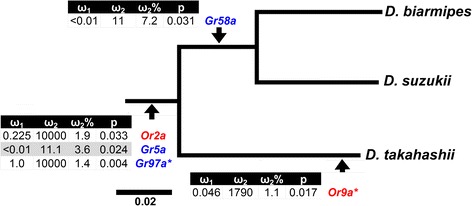
Genes with signatures of positive selection in the *suzukii-takahashii* clade based on the adaptive branch-site random effects likelihood (aBSREL) method [52] performed across 59 *Or* and 61 *Gr* homologous gene sets within a phylogenetic framework comprised of nine *melanogaster* group *Drosophila*. P-values were corrected for multiple tests within each gene set using the Holm-Bonferroni method [63]. *Genes also showing signatures of positive selection using the branch site method in PAML [65]

Next we tested for the signatures of positive selection acting on a small proportion of sites that are often difficult to detect using the *dN*/*dS* ratio across the entire gene. The adaptive branch-site random effects likelihood (aBSREL) approach [52] on homologous gene sets revealed two *Ors* and three *Grs* showing evidence of positive selection the *suzukii-takahashii* clade in the phylogenetic framework comprising nine *melanogaster* group drosophilids (Fig. 5; Additional file 2, Table S5). The number of tests for each gene set ranged from a small set of 11 (singletons with losses in some lineages) to as many as 43 for a gene with large expansion across spp. (*Gr59d*). A total of five lineages showed signatures of positive selection, four of those being at internal nodes and one being at an external node (Fig. 5). In all cases, the percentage of sites exhibiting signatures of positive selection (ω_2_%) was small, ranging from 1.1 % to 7.2 % (Fig. 5). Selection at the remaining sites (ω_1_) ranged from very high purifying selection (ω_1_ < 0.01) to neutral selection (ω_1_ = 1).

In the *suzukii*-*takahashii* clade, positive selection was detected in *Or2a*, *Gr5a* and *Gr97a* along branches leading to both the *suzukii* and *takahashii* subgroups, while *Gr58a* showed signatures of positive selection along the branch leading to the *suzukii* subgroup (Fig. 5). In *Or2a*, positive selection was found at a very small percentage of codons (1.9 %), while the remaining sites exhibit signatures of purifying selection (ω_1_ = 0.225) (Fig. 5). Strong purifying selection (ω_1_ < 0.01) was evident at 96.4 % of *Gr5a*, while 3.6 % of the codons showed evidence of positive selection (ω_2_ = 11; *p* = 0.024). *Gr58a* also exhibited strong purifying selection (ω_1_ < 0.01) at the majority of sites (92.8 %), while the remaining 7.2 % exhibited signatures of positive selection (; *p* = 0.031). The vast proportion of *Gr97a* (98.6 %) shows no signs of selection pressure (ω_1_ = 1.0) while 1.6 % of the sites show evidence for positive selection (*p* = 0.004).

**Fig. 6:**
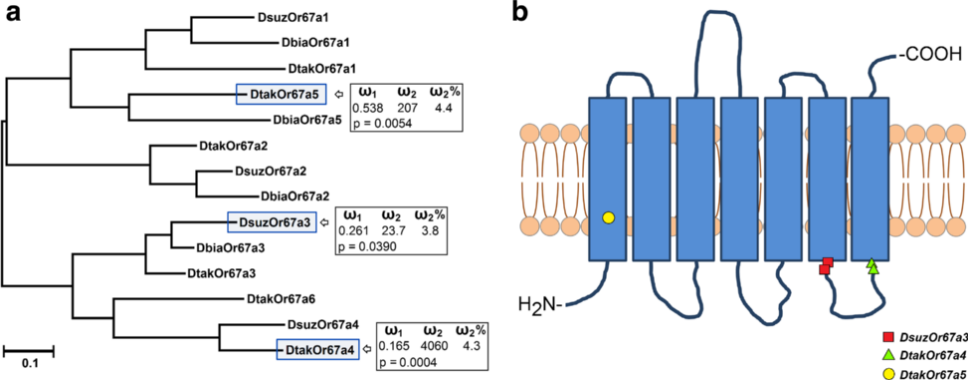
Signatures of positive selection in the suzukii-takahashii Or67a lineage. **a** Three gene showed signatures of positive selection in the highly expanded Or67a lineage. Phylogeny adapted from Chiu et al. [35]. **b** The approximate position of the sites under positive selection based on branch site tests in PAML and the transmembrane domain predictions by OCTOPUS

Next, the two largest expanded *Or* and *Gr* lineages were subjected to aBSREL analysis by restricting the phylogeny to the three species in the *takahashii-suzukii* clade. Only the *Or67a* lineage had genes with signatures of positive selection, of which two genes were in *D. takahashii* (*DtakOr67a-4* and *DtakOr67a-4*) and one in *D. suzukii* (*DsuzOr67a-3*) (Fig. 6a). These results were independently confirmed using the branch-site test in PAML that further identified codons under positive selection (Fig. 6b).

Finally, we would like to state that the reason for reporting the less stringent p-values from Holm-Bonferroni corrections within gene sets was to extract candidates with some (any) evidence of positive selection.

## Discussion

### Olfactory structures

Peripheral olfactory structures in *D. melanogaster* have been studied over the years and have revealed stereotypic pattern of sensillary organization [6, 71, 73]. These studies laid a solid foundation to the functional mapping of sensilla [74, 75]. More advanced molecular techniques have correlated the morphological and functional sensillary patterns with that of chemosensory gene expression [76, 77]. A broadly conserved pattern emerged in our *D. suzukii* SEM studies as compared to *D. melanogaster*. Limited single sensillum recordings (SSR) from *D. suzukii* antennal basiconic (ab) sensilla in combination with high resolution gas chromatography (GC-SSR) suggested a high conservation in response profile from the ab1 sensilla as compared to *D. melanogaster*, whereas other two large sensilla (ab2 and ab3) had significantly altered physiological profiles [39]. This could be due to the alteration in *Or* sequences and/or expression profiles.

### Repertoire size

Unlike vertebrates and many insects, in which there has been extensive variation in the number of genes in the chemoreceptor families, the size of the *Or* and *Gr* repertoires in *Drosophila* have changed little during the last ~70 million years [78] despite their extensive distribution and diverse life history traits [79] that range from primitive sap and slime feeding (*virilis-repleta*) to more recent adaptations in the *melanogaster* group that utilize decaying and fermenting fruits [26]. Of ~30 *Drosophila* genomes that have been sequenced, the *melanogaster* group is the most represented. Furthermore, this group has been well characterized in terms of chemosensory repertoire annotation.

A number of previous studies have described a balanced birth-and-death process of evolution, wherein the number of genes gained through duplication roughly equals the number of genes lost through pseudogenization, thus maintaining *Or* and *Gr* repertoires comprising approximately 60 genes each [5, 21, 37, 80]. Our chemoreceptor annotations in the *suzukii*-*takahashii* clade revealed similar patterns (Fig. 4; Additional file 2: Tables S3 and S4). The size of the *Or* repertoires in *D. suzukii* and *D. biarmipes* are roughly the same as other *melanogaster* group flies, while *D. takahashii* has several additional *Ors* that make its repertoire the largest among all the annotated species in this group with 77 intact Ors. The size of the *Gr* repertoires in *D. suzukii*, *D. biarmipes* and *D. takahashii* are all relatively large compared to other *melanogaster* group members, with 81, 83 and 91 total proteins, respectively. Analysis of the evolutionary history of duplications and losses revealed that the expansions of the *Gr* lineages in the *suzukii*-*takahashii* clade occurred prior to *D. suzukii’s* divergence from *D. biarmipes,* ~7.3 mya [36]. Thus, the expanded *Gr* lineage is not a direct consequence of *D. suzukii’s* adaptation to its expanded ecological niche, but could have simply helped facilitate the shift by providing ample variation for evolution to act upon.

### Expansions and losses

Despite the maintenance of a standard repertoire size, gene births and deaths during trophic shifts can produce unique and rapidly evolving chemosensory repertoires. A study by McBride [19] showed that *D. sechellia,* a species endemic to the Seychelles and a specialist on the fruit of *Morinda citrifolia,* experienced an accelerated rate of chemoreceptor gene loss during its evolution to a specialist life style. A similar trend in the *Grs* was found in *D. erecta*, a specialist on *Pandanus candelabrum* [20]. A recent study further demonstrated a relationship between host-choice and chemoreceptor repertoire wherein four widely conserved *Ors* (*Or9a, Or22a, Or42b and Or85d*) that detect yeast-derived and fruit related compounds were uniquely lost in an herbivorous *Drosophila*, *Scaptomyza flava*, while *Or67b*, a receptor shown to enhance the sensitivity and detection of plant derived green leaf volatiles, was uniquely expanded [18]. These unique changes in the *Or* repertoire were considered as adaptive losses and gains towards the evolution of herbivory in *Scaptomyza* from its ancestral drosophilids that feed on yeast [18, 81].

The *D. suzukii* and *D. takahashii Or* repertoires are distinct in having two large expansions, *Or23a* and *Or67a*, while only the *Or67a* expansion was retained in *D. biarmipes.* In *D. melanogaster*, *Or23a* is expressed on the surface of the B cell in antennal intermediate 2 (ai2) sensilla [82], formerly classified as antennal trichoid 2 (at2) sensilla [76]. And despite screening with a large panel of compounds using SSRs and the *Δ-halo* system in *D. melanogaster*, no strong ligands for *Or23a* have been identified [83, 84]. In *D. melanogaster*, *Or67a* is expressed on the surface of the B cell in ab10 sensilla (Couto et al. 2005), where methyl benzoate and ethyl benzoate elicited strong excitatory responses (≥100 spikes/s) at a low dose of 10^−4^ dilutions [83]. Five functional copies of *Or67a* in the *D. suzukii* strain from Italy have been found [40], while we identified only four intact copies and one pseudogene in the North American isolate, suggesting that the number of functional genes in the *Or67a* lineage can be variable across geographical regions. This group also suggested that *D. suzukii’s* increased sensitivity to isoamyl acetate [33, 39], a yeast-derived and fresh fruit volatile, could be due to the expanded *Or67a* copy-numbers [40].

Interestingly, of the three species annotated here, *D. suzukii’s* repertoire of *Ors* underwent the most gene deaths, with losses of *Or74a*, *Or85a* and *Or98b*. This results in the smallest number of *Or* lineages (51) among the nine drosophilids studied here (Additional file 2: Table S3). It is worth mentioning that this number of lineages is even smaller than *D. sechellia’s* and *D. erecta’s*, both of which have a very restricted diet. Of the three lost lineages, *Or74a* in *D. melanogaster* is a larval specific receptor expressed in a sub-set of ORNs in the larval dorsal organ (LDO) [85, 86] (Table 4). A heterologous expression using *Δ-halo* system revealed excitatory responses to linear aliphatic compounds such as 1-hexanol, (*E*)-2-hexenal, 1-heptanol and 1-nonanol (≥100 spikes/s), compounds commonly associated with fruits [86] (Table 4). The second, *Or85a*, is a narrowly tuned receptor expressed on the B cell of ab2 sensilla in *D. melanogaster* where ethyl 3-hydroxybutyrate elicits a strong excitatory response [87]. Single sensillum recordings (SSRs) by Keesey et al. [39] showed similar response profiles for the B cell in ab2 sensilla in *D. biarmipes* and *D. melanogaster*, but not for *D. suzukii*. Ethyl 3-hydroxybutyrate still elicited a strong response, but 2-heptanone elicited the strongest response in *D. suzukii* (Table 4). However, 2-heptanone did not elicit a response in *D. biarmipes* or *D. melanogaster*, suggesting that a different, more broadly tuned Or is being expressed in *D. suzukii’s* ab2 sensillum, which lends physiological evidence for the loss of *Or85a* from *D. suzukii’s* repertoire of functional *Ors*. Very little is known about the function of *Or98b*, except for its co-expression with *Or85b* in the A cell of the ab6 sensillum in *D. melanogaster* [76].

**Table 4:** Ligands and chemosensory organs, based on studies in *D. melanogaster*, are shown for lost and expanded lineages, and genes with signatures of positive selection (a = antenna, *p* = palp, b = basiconic, t = trichoid, LDO = larval dorsal organ)

	Gene	Species	Ligands	Expression
Losses	*Or33cP*	bia	Ethyl acetate, Cyclohexanone, Fenchone [100]	pb2A [76, 100]
	*Or74aP*	suz	*E,E*-2-4-nonadienal [101]	LDO [86]
	*Or85aP*	suz	Ethyl 3-hydroxybutyrate [87]	ab2B [76]
	*Or98bP*	suz	unknown	ab6B*[76]
Expansions	*Or23a*	suz, tak	Isoamyl acetate [83]	ai2B [76]
	*Or67a*	suz, bia, tak	Ethyl benzoate, Methyl benzoate [83]	ab10B [76]
	*Gr22a-f*	tak	bitter compounds [102]	Labellum [103], larvae [104] legs [105]
	*Gr36a-c*	suz, bia, tak	bitter compounds [102]	Larvae [104], legs [105]
	*Gr59ab*	suz, bia, tak	bitter compounds [102]	Larvae [104], legs [105]
	*Gr59cd*	suz, bia, tak	bitter compounds [102]	Larvae n [104], legs [105]
	*Gr93a*	suz	bitter compounds [102]	unknown
	*Gr98b*	suz	bitter compounds [102]	unknown
	*Gr98d*	suz	bitter compounds [102]	Legs [105]
Positive sel.	*Or2a*	suz, bia, tak	Ethyl 3-hydroxybutyrate, Isoamyl acetate [83]	ai3A [76]
	*Or9a*	tak	2-acetoin, 2,3-butanediol [84]	ab8B [76]
	*Gr5a*	suz, bia, tak	Trehalose [93, 94]	Labellum [103], legs [105]
	*Gr58a*	suz, bia	unknown	unknown
	*Gr97a*	suz, bia, tak	unknown	Larvae [104]

*Not confirmed

Finally, we made numerous attempts to sequence all three lost lineages in the *D. suzukii* genome. Our sequencing of *Or74a* and *Or85a* confirmed the highly degraded state of the loci in the North American isolate [35]. However, these two genes were considerably less degraded in the genome assembly from the Italian isolate, but pseudogenizations were still apparent [36]. Conversely, we were unsuccessful in sequencing the *Or98b* locus in *D. suzukii*. Amplicon size was consistent with that of a full length gene, but sequencing indicated that the locus is polymorphic in the North American assembly. However, we were able to build an intact gene model for *Or98b* in the genome assembly from the Italian assembly [36], and that sequence is provided in Additional file 1, Table S1. Polymorphism in *Or98b* among *D. melanogaster* strains was also reported, wherein several functional and pseudogene alleles were found in the Ives strain, a single pseudogene was found in the New Jersey strain, and no allele could not be amplified in the Oregon R strain [5].

### Divergence

Measures of divergence provide insights into the molecular evolution which can often be correlated with conserved and divergent physiological processes. Our measure of divergence (*dN*/*dS)* implies that both chemoreceptor families have evolved under strong purifying selection. However, these values are larger than the genome wide estimates of 0.095 for X chromosome genes and 0.090 for autosomal genes [35], demonstrating that these gene families are more divergent than average. Comparisons between *dN*, *dS*, and *dN/dS* of *Ors* and *Grs* using unpaired tests showed that *Grs* are more divergent than *Ors* in all three species (Table 3).

Among the most conserved Ors, *Orco* tops the list, followed by *Or47a*, *Or92a*, *Or42b* and *Or24a*. These genes are also highly conserved in *D. takahashii* and *D. biarmipes.* Expression studies in *D. melanogaster* have revealed *Orco* to be a non-canonical receptor with a wide distribution [9, 10, 77], whereas expression of the remaining *Ors* is confined to basiconic sensilla [76] except for *Or24a* which is larval specific in *D. melanogaster* [85]. Interestingly *Or92a* and *Or42b* are expressed in ab1 sensilla on the A and B ORNs, respectively. This high level of conservation corresponds with the electrophysiological data of ab1 that showed similar responses to a panel of ab1-sensitive odorants in *D. melanogaster*, *D. biarmipes* and *D. suzukii* [39]. An earlier study showed similar findings comparing nine species in the *melanogaster* subgroup [24]. Combined, these findings suggest that the role of ab1 sensilla has largely been conserved during at least the last ~13 million years of *melanogaster* group evolution. In fact, McBride and Arguello [20] proposed this phenomenon to be applicable for all the large basiconic sensilla (ab1-3) in five members of the *melanogaster* subgroup.

On the other hand, the expression of the most divergent receptors in *D. suzukii* is predicted to be among three different sensilla types. Of these, both *Or19a* and *Or23a* are expressed in intermediate sensilla [71, 82], *Or33a* and *Or69aA* are restricted to a basiconic [71, 82], and *Or65a* is expressed in a trichoid [76]. Potential response characteristic and the significance of these *Ors* in *D. suzukii* remains an exciting avenue to explore. Three of these five homologues in *D. melanogaster* (*Or23a*, *Or65a*, and *Or69aA*) did not respond with high sensitivity to any of the odorants tested heterologously [83]. Physiological data is lacking for *DmelOr33a*. Two different studies reported *DmelOr19a* responding to limonene, a major citrus fruit volatile [83, 88].

Among the gustatory receptors in the *suzukii-takahashii* clade, *Gr21a* was the most conserved, surpassing even *Orco*. The other highly conserved *Grs* include *Gr28bB*, *Gr28a*, *Gr63a* and *Gr64c*. It is worth mentioning that *Gr21a* and *Gr63a* are highly conserved among insects [5, 80], and together confer the sensitivity to carbon dioxide [89, 90], whereas *Gr28bB* and *Gr28a* are part of the bitter receptor family and are shown to be ubiquitously expressed in a wide array of sensory and non-sensory tissue [91, 92]. The five most divergent *Grs* include *Gr10b*, *Gr93d*, *Gr92a*, *Gr85a* and *Gr22c*; little is known about their expression or response characteristics.

### Selection

Our set of 11 chemoreceptor lineages with signatures of positive selection in the nine species is smaller than the reported 20 in an earlier study that compared chemosensory repertoires in 12 *Drosophila,* even though two genes (*Or9a* and *Gr5a*) were common in both studies [17]. These differences could be due to multiple reasons. Our study focused on the drosophilids from the *melanogaster* group that have a relatively comparable host range [26], while the other study included six species outside the *melanogaster* group. In addition, we adjusted the *p*-values based on more stringent Holm-Bonferroni corrections which reduced the number of significant candidates. However, we note that our corrections were performed within, but not across gene sets; therefore, these results should be interpreted with caution.

Of the 11 genes, we found four genes (*Or2a*, *Gr5a*, *Gr58a* and *Gr97a*) that were significant in the branches leading to *D. suzukii*. In *D. melanogaster*, *Or2a* is expressed in ai3 sensilla [76, 82] and has been shown to respond to ethyl 3-hydroxybutyrate and isoamyl acetate eliciting only moderate responses (~50 spikes/s) [83]. It is interesting to note that isoamyl acetate has been identified as a strong ligand from *suzukii*-associated yeasts [33] and host fruits [40]. Among the *Grs*, *DmelGr5* has been studied in detail. Molecular, physiological and behavioral studies identified it as a sugar receptor with a strong selectivity and sensitivity to trehalose [93, 94]. Importance of sugars in *D. suzukii* is more pronounced since this fly also uses a variety of non-conventional sugar sources such as nectar and cherry blossom in the field [95]. Functional data on *DmelGr58a* and *DmelGr97a* is lacking [91]. Our restricted aBSREL analysis of the four largest expanded lineages (*Or23a*, *Or67a*, *Gr59a* and *Gr59d*) in the *suzukii-takahashii* clade revealed evidence for positive selection only in *Or67a*, where three copies showed signatures of positive selection (Fig. 6a). Overall, adaptation of *D. suzukii* to novel niches appears to be facilitated by unique expansions and losses of chemosensory lineages. Together with our earlier that described the volatile chemical landscapes of *D. suzukii* [33], present study further provides novel insights into the synchronous evolution of signaling and reception in flies.

## Conclusions

We manually annotated the olfactory and gustatory receptor families of the pest fly, *D. suzukii* to complement our earlier analysis of the evolution of olfactory signals in this fly that showed salience of a set of yeast derived odorants enriched in the *D. suzukii* landscape [33]. We further annotated two close relatives, *D. biarmipes* and *D. takahashii* to compare and contrast their chemosensory repertoire with that of *D. suzukii*. This revealed three unique losses of *Ors* (*Or74a*, *Or85*, *Or98b*) in *D. suzukii* among the three species in the *suzukii-takahashii* clade, and two large expansions in the olfactory receptors, *Or23a* and *Or67a*. There was an overall pattern of purifying selection in both chemoreceptor families, with *Ors* exhibiting greater conservation. The gustatory genome repertoire size in this clade was by far the largest among all the annotated species of the *melanogaster* group. Finally, our analysis for the signature of positive episodic selection in *D. suzukii* led to the identification of *Or2a* and one copy of *Or67a* as strong candidates. Taken together, this study provides detailed insights into the molecular evolution of the two major chemoreceptor families in an invasive and pestiferous fly. The evolution of a serrated ovipositor for piercing the skin of fresh fruits is a unique innovation that conferred a distinct advantage in fruit flies to exploit fruits of varying ripeness. In tephritids, this innovation facilitated the radiation of thousands of species [96]. Surprisingly, this innovation exists in only two known drosophilids, *D. suzukii* and *D. subpulchrella*, both of which are members of the *suzukii* subgroup [31]. The recent sequencing of *D. suzukii* (pest) and *D. biarmipes* (non-pest) within the *suzukii* subgroup provided us with an excellent opportunity to explore the contribution of chemosensation in the evolution of pestilence in *D. suzukii*.

## Abbreviations

aBSREL, adaptive branch-site random effects likelihood; *Gr*, gustatory receptor; LRT, likelihood ratio tests; *Or*, odorant receptor; *Orco*, olfactory receptor co-receptor; ORN, olfactory receptor neuron; SEM, scanning electron microscopy; VOC, volatile organic compounds

## Additional files

Additional file 1: Table S1.
*D. suzukii* Or coding and peptide sequences. **Table S2.** *D. suzukii* Gr coding and peptide sequences. **Table S3**. *D. biarmipes* Or coding and peptide sequences. **Table S4.** *D. biarmipes* Gr coding and peptide sequences. **Table S5.** *D. takahashii* Or coding and peptide sequences. **Table S6.** *D. takahashii* Gr coding and peptide sequences. (XLSX 379 kb) Additional file 2: Table S1. Non-synonymous (*dN*) and synonymous (*dS*) substation rates and ratios (*dN*/*dS*) of *Ors* in members of the *suzukii-takahashii* clade using *D. melanogaster* as an outgroup. **Table S2.** Non-synonymous (*dN*) and synonymous (*dS*) substation rates and ratios (*dN*/*dS*) of *Grs* in members of the *suzukii-takahashii* clade using *D. melanogaster* as an outgroup. **Table S3.** The number of functional *Or* genes in each orthologous group for nine species in the *melanogaster* group based on Almeida et al. [50]. **Table S4.** The number of functional *Gr* genes in each orthologous group for nine species in the *melanogaster* group based on Almeida et al. [50]. **Table S5.** Summary of the chemoreceptors in the *suzukii-takahashii* clade under episodic positive selection as revealed by aBSREL analyses. (XLSX 35 kb) Additional file 3: Or alignment file (Phylip). Olfactory receptor peptide sequences of *Drosophila suzukii* (Dsuz), *D. biarmipes* (Dbia), *D. takahashii* (Dtak) and *D. melanogaster* (Dmel) ≥ 360 aa in length were multiply aligned using MUSCLE v3.8.31. (PHY 229 kb) Additional file 4: Or phylogenetic tree file (Nexus). Phylogenetic analysis of Ors in *Drosophila suzukii* (Dsuz), *D. biarmipes* (Dbia), *D. takahashii* (Dtak) and *D. melanogaster* (Dmel) using a Maximum Likelihood method. Evolutionary history was inferred using a Maximum Likelihood method based on the JTT matrix-based model. The tree was constructed using RAxML under the JTT model of substitution with NNI topology search, based on an amino acid alignment by MUSCLE. Branch support was estimated using 500 bootstrap replications. The tree is rooted with *Orco*. (NEX 26 kb) Additional file 5: Gr alignment file (Phylip). Gustatory receptor peptide sequences of *Drosophila suzukii* (Dsuz), *D. biarmipes* (Dbia), *D. takahashii* (Dtak) and *D. melanogaster* (Dmel) ≥ 340 aa in length were multiply aligned using MUSCLE v3.8.31. (PHY 274 kb) Additional file 6: Gr phylogenetic tree file (Nexus). Phylogenetic analysis of Grs in *Drosophila suzukii* (Dsuz), *D. biarmipes* (Dbia), *D. takahashii* (Dtak) and *D. melanogaster* (Dmel) using a Maximum Likelihood method. Evolutionary history was inferred using a Maximum Likelihood method based on the JTT matrix-based model. The tree was constructed using RAxML under the JTT model of substitution with NNI topology search, based on an amino acid alignment by MUSCLE. Branch support was estimated using 500 bootstrap replications. The tree is rooted with *Gr21a*. (NEX 31 kb) Additional file 7: Figure S1. Evolutionary history of duplications and losses in four expanded lineages in the chemoreceptor families based on the parsimony-based method of gene tree reconciliation in NOTUNG v2.8.1.6 [51]. (TIF 2581 kb)
