# Supplementary figures and images for: The making of a pest: Insights from the evolution of chemosensory receptor families in a pestiferous and invasive fly, Drosophila suzukii
Source: BMC Genomics. 2016 Aug 17;17:648. doi: 10.1186/s12864-016-2983-9 (PMC4988008; doi:10.1186/s12864-016-2983-9)

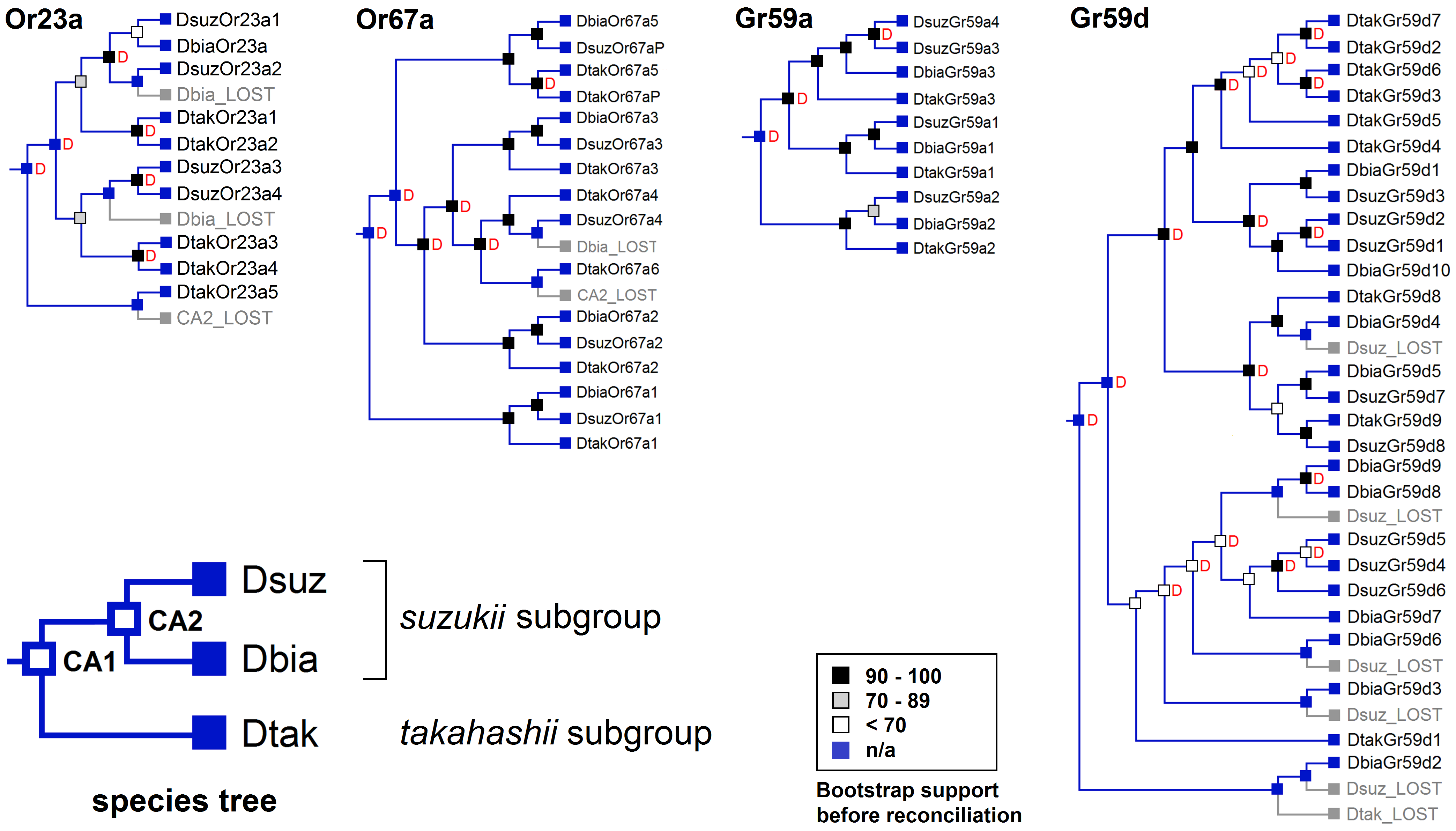

Supplement: Additional file 7: Figure S1. — Evolutionary history of duplications and losses in four expanded lineages in the chemoreceptor families based on the parsimony-based method of gene tree reconciliation in NOTUNG v2.8.1.6 [51]. (TIF 2581 kb) [file 12864_2016_2983_MOESM7_ESM.tif]
